# Testing the Multiple Disadvantage Model of Health with Ethnic Asian Children: A Secondary Data Analysis

**DOI:** 10.3390/ijerph20010483

**Published:** 2022-12-28

**Authors:** Tyrone C. Cheng, Celia C. Lo

**Affiliations:** 1 School of Social Work, University of Alabama, Little Hall, Tuscaloosa, AL 35401, USA; 2 Behavioral Research Manager, Peraton, Defense Personnel and Security Research Center, Seaside, CA 93955, USA

**Keywords:** Asian children, health, multiple disadvantages, welfare

## Abstract

This study of ethnic Asian children in the United States asked whether their health exhibited relationship with any of six factors: social disorganization, social structural factors, social relationships, the health of their parents, their access to medical insurance, acculturation. The sample of 1350 ethnic Asian children was extracted from the 2018 National Survey of Children’s Health. Logistic regression results showed that these children’s excellent/very good/good health was associated positively with safe neighborhoods, family incomes, family cohesiveness, family support, and receipt of Temporary Assistance for Needy Families (TANF). In turn, health was associated negatively with single-mother households. Implications of the present results in terms of interventions promoting family support, TANF participation, safe neighborhoods, and professionals’ cultural competency are discussed.

## 1. Introduction

Research results have long suggested that, in the United States, child health is associated negatively with minority ethnicity [1,2,3,4,5]. Some studies, nevertheless, have reported anywhere from 78.4% to 96.5% of ethnic Asian children surveyed to have health described as excellent/very good/good [6,7,8,9]. In contrast, two published studies found health among ethnic Asian children to be worse than health among white children [10,11], and several studies found no significant differences in health between ethnic Asian children and white children [7,12,13,14]. It is important, then, to continue examining factors that may be involved in the health of ethnic Asian children.

### 1.1. Multiple Disadvantage Model

To conduct its investigation of factors in ethnic Asian youngsters’ health, the present study applied a theoretical model—the multiple disadvantage model. This model holds that socioeconomic disadvantages and the distress associated with them negatively affect intimate relationships and social relationships. It has been applied to explain children’s health as related to maternal health [15]; to explain children’s delinquency [16,17,18]; to explain access to substance use treatment [19]; and to explain racial disparities in victimization [20,21,22]. The multiple disadvantage model deems historical and structural racism to continually frustrate members of our society who are of minority ethnicity [20]. If these individuals are parents, racism-related frustration may, the model predicts, exhibit a negative influence on their parenting. The literature does offer at least one analysis, though, arguing that to date the search for a racism–health link among children generally (that is, children of all ethnicities) has yielded mixed results [23].

#### 1.1.1. Social Disorganization

**Figure 1:**
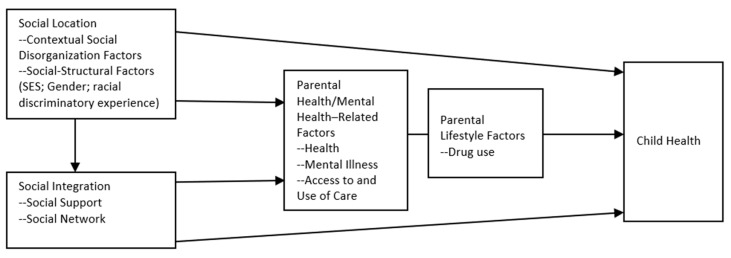
The multiple disadvantage model explaining Asian child health.

In the present study, we applied the multiple disadvantage model to examine how Asian child health might be related to five socioeconomic disadvantages: social disorganization, social structural factors, social relationships, parental health, and medical insurance (see Figure 1). The presence of socioeconomic disadvantages tends to impair both individual physical health and parenting, leading to poor health for children [15]. Prior studies with children in the general population have shown poor health to be linked to social disorganization, for instance to low income and to living in unkempt, unsafe neighborhoods [1,3,24,25,26,27,28,29]. One study reported a link between ethnic Asian children’s poor health, specifically, and the disorganized or materially deprived condition of neighborhoods [30]. For the present study, we speculated that social disorganization factors similar to these are related to health among ethnic Asian children.

#### 1.1.2. Social Structural Factors

We speculated that social structural factors—namely, parents’ education, employment status, and income—are, when their attainments are low, a considerable deterrent to the good or excellent health of ethnic Asian children. However, the literature suggests that ethnic Asian children’s health is not significantly related to parents’ educational attainment [31,32]. On the other hand, research with children of all ethnicities has reported child health to be associated positively with current employment of parents [33,34] and with parents’ income [2,3,4,13,35,36,37]. While one study reported that receiving public assistance from Temporary Assistance for Needy Families (TANF) or the Supplemental Nutrition Assistance Program (SNAP) is associated positively with children’s health [38], another showed a negative association [39]. We ultimately speculated that the health of Asian children would be linked positively to their parents’ education, income, and current employment.

#### 1.1.3. Social Relationships

Supportive social relationships can alleviate the distress of Asian parents facing multiple socioeconomic disadvantages. Parents who are supported by strong social networks demonstrate relatively more-effective parenting, according to the literature [40], and that in turn eventually improves children’s health [27,41,42]. Two prior studies with a general population of children found poor health to be associated with single-parent families and with parents’ separation [36,43]; other studies, however, observed no health-related association either for single-parent households or for support from parents’ partner/former partner [4,44]. Additionally, a study in the literature reported no significant relationship between Asian ethnicity and the degree or quality of parents’ communication with children’s healthcare providers [45]. As well, one published study interestingly reported ethnic Asian children to have received less emotional support from their parents than white children received from theirs [46]. For the present study, we speculated that health among ethnic Asian children would be associated negatively with single-parent households but associated positively with family and social support.

#### 1.1.4. Parental Health

The multiple disadvantage model proposes that facing socioeconomic disadvantages can affect physical health. The literature appears not to address the specific disadvantage of interest to our present study, which would be the physical health of parents of ethnic Asian children. However, studies with a general population of children have indicated that parents in good physical health care relatively effectively for their children [47]. Moreover, published studies also indicate that parents’ physical health is associated positively with their children’s physical health [15,35,39,48,49]. For this study, we speculated that a positive relationship would be observed between the health of ethnic Asian children and their parents’ own health.

#### 1.1.5. Medical Insurance

Lack of medical insurance was the fifth socioeconomic disadvantage affecting families included in our present study. Lack of health coverage can affect children’s health, uninsured children reportedly being less healthy than insured children [3,48,49]. For this study, we speculated that a positive relationship would be observed between health among ethnic Asian children and their coverage by medical insurance.

### 1.2. Acculturation

Another factor we tested as a potential indicator of ethnic Asian children’s health was acculturation. Acculturation is the process through which individuals and groups adjust to and/or are changed by a culture beyond their native culture [50], typically as a result of immigration. For Asian individuals and families in the U.S., proficient spoken English is an indicator of acculturation [51]. Studies of ethnic Asian children have concluded that their physical health is associated positively with their English language learning [8,9,52] and moreover is associated negatively with their and/or their parents’ having immigrated to the U.S. [6,53,54]. According to one study—and not surprisingly—only 43% of surveyed first-generation Asian immigrants to the U.S. demonstrated proficiency with English [55]. A different study reported finding no significant association between the health of ethnic Asian children and such children’s own or their parents’ nativity [32].

### 1.3. Hypotheses

Along with acculturation’s role, the present study probed, again, whether facing multiple socioeconomic disadvantages hinders parental/family caregiving for ethnic Asian children, and whether any effect on caregiving seems in turn to diminish children’s health. The reviewed literature provided only a small number of studies focusing on factors in the health of ethnic Asian children in the U.S. Based on that modest beginning and applying the multiple disadvantage model, we devised two hypotheses for testing, as follows:

1. Health among ethnic Asian children will be associated positively with safe neighborhood, family income, parents’ educational attainment, parents’ employment, family support, social support, parents’ health, medical coverage, TANF or SNAP enrollment, parents’ birth in U.S., children’s birth in U.S., and families’ English proficiency.
2. Health among ethnic Asian children will be associated negatively with rundown neighborhood, discrimination experience, and single-mother household.

## 2. Methods

### 2.1. Sample

This secondary data analysis employed a nationally representative sample of 1350 ethnic Asian children extracted from a public-use data set, the 2018 National Survey of Children’s Health (NSCH, Salem, MA, USA). NSCH researchers interviewed 30,530 children and their caregivers, gathering information on health status, insurance coverage, social relationships, family relationships, and neighborhood characteristics [56]. Our sample was limited to participating children of Asian ethnicity and their parents. In our present sample, child’s median age was 10 years, and girls constituted nearly 51% of our sample. This study used a public-use data set and was exempted from approval by a university Institutional Review Board.

### 2.2. Measures

Our outcome variable, child health, was dichotomized as “excellent/very good/good” versus “fair/poor,” the latter serving as the reference. In the original NSCH study, participants had been offered the responses “excellent,” “very good,” “good,” “fair,” and “poor.” Our explanatory variables made up seven groups: social disorganization factors, social structural factors, social relationships and social support, parental health, medical insurance, acculturation factors, and demographic characteristics.

Our social disorganization factors comprised two dichotomous variables and one continuous variable. Rundown neighborhood indicated that a parent had (yes) or had not (no) reported his/her neighborhood (a) to have “litter or garbage on street or sidewalk,” (b) to feature “poorly kept or rundown housing,” or (c) to feature “vandalism such as broken windows or graffiti.” Racial discrimination (yes/no) measured whether his/her parent had reported a participating child to have ever been treated or judged solely on race/ethnicity. Safe neighborhood described how much a participating child’s parent agreed that the family’s neighborhood was safe for the child, using the offered responses 4 (definitely agree), 3 (somewhat agree), 2 (somewhat disagree), and 1 (definitely disagree).

Our social structural factors included variables measuring parents’ educational attainment as well as family income. Parent educational attainment gave the highest level of study completed, using offered responses as follows: 1 (8th grade or below), 2 (9th–12th grade), 3 (graduated high school or GED), 4 (vocational school), 5 (some college), 6 (associate degree), 7 (undergraduate degree), 8 (master’s degree), 9 (doctoral or professional degree). Employed parent (yes/no) described parents who had been paid employees during 50 of the 52 weeks preceding NSCH interviews. Family income-to-poverty ratio gave the percentage of federal poverty level that a family’s income represented, figures provided in the NSCH data set. Finally, participation in public assistance programs was measured via two variables, receipt of TANF and receipt of SNAP, describing families’ receipt of associated benefits during the 12 months preceding interview.

We used six explanatory variables to measure social relationships and social support. Single mother (yes/no) described parents who were single female parents. Next, a response scale was used to measure family cohesiveness and involved two survey items. Parents were asked whether their families drew on strengths that family members possessed, and they were asked whether their families talked together about problems they faced. The response scale comprised 1 (none of the time), 2 (some of the time), 3 (most of the time), and 4 (all the time). Scores for the two items were summed to obtain a total score for each parent, with a higher total score indicating stronger family cohesiveness. The measure yielded a Cronbach’s alpha of 0.85.

In addition, we used the dichotomous variable family support to indicate whether a spouse/partner, other family members, or friends were providing a parent with emotional support encouraging his/her parenting efforts. Similarly, the dichotomous variable professional support indicated whether a counselor or other healthcare provider was supplying a surveyed parent with emotional support, and another dichotomous variable peer/religious group support indicated whether a parent had joined a support group of peers or a religious group for the purpose of obtaining emotional support. Finally, neighbor support was measured via a total score from survey items asking parents how much they agreed that adults in the neighborhood (a) know where to get help, (b) watched out for each other’s children, and (c) provided help to other parents when requested to. A relatively high total score implied a relatively strong network of supportive neighbors. For all three items, offered responses were 4 (definitely agree), 3 (somewhat agree), 2 (somewhat disagree), and 1 (definitely disagree). The three items yielded a Cronbach’s alpha of 0.79.

Resembling our child health outcome variable, our explanatory variable parent health was dichotomized as “excellent/very good/good” versus “fair/poor”, the latter serving as the reference. Parent health was a self-reported measure. Our study also considered some variables describing families’ participation in public or private medical insurance or assistance programs. Preliminary analysis of a variable indicating private insurance participation, however, suggested the dichotomous variable was vulnerable to singularity, due to our preliminary modeling’s inability to estimate the variable’s coefficient, or odds ratio. The coefficient could not be estimated because we encountered no cases in which a parent holding private insurance reported a child to be in “fair/poor” health. In light of the real possibility of singularity, our final analysis employed a single dichotomous variable, insured, to indicate a child’s coverage by either public or private medical insurance.

We used three dichotomous explanatory variables to measure a family’s acculturation: parent born in U.S., child born in U.S., and speaks English at home. The latter variable stated whether or not English was the language largely used in the family’s home. We did not include parent’s U.S. residence less than five years as a variable because doing so generated singularity in preliminary analysis. Finally, we used three demographic variables as controls in our modeling. The three were parent age (in years), child age (in years), and girl (boy providing the reference).

### 2.3. Data Analysis

In preparing descriptive statistics, we analyzed categorical variables using frequencies and percentages. Since continuous variables were found not normally distributed, they were analyzed by using medians and interquartile ranges. Because our present study’s outcome variable was a binary one, we employed STATA logistic regression to perform linearized variance estimations with robust standard errors, and we added all explanatory variables to the logistic regression model at the same time. In addition, we employed the sampling weights that the NSCH researchers had provided. Our preliminary analyses of tolerance statistics (0.51 or higher) and of correlations (−0.28 ≤ *r* ≤ 0.65) suggested no multicollinearity problems among the employed explanatory variables.

## 3. Results

Descriptive analyses demonstrated that a great majority (98.6%) of the ethnic Asian children to have “excellent/very good/good” health (see Table 1). Descriptive statistics also showed 25.9% of these children to live in rundown neighborhoods and 6.2% to have experienced racial discrimination. The median score for safe neighborhood was 4 (i.e., parents reported themselves to “somewhat agree” that their neighborhoods were safe for their children). In this study, the parent’s median educational attainment measured 7, or earning of an associate’s degree. On average in the study, a family had a median income-to-poverty ratio of 390%. Nearly 82.0% of parents in our study were employed; only 1.2% had received TANF assistance, and 4.3% had received SNAP assistance.

Out of parents of 1350 ethnic Asian children in the present sample, only 5.7% were single mothers. The median score for family cohesiveness was 7 and for neighbor support was 10. Of our sample, 51.9% reported receiving emotional support from members of the family, and 16.4% reported receiving emotional support from healthcare professionals. For 18.9% of parents in our sample, emotional support was provided by a peer/religious group. In addition, 96.5% of parents in our sample reported having “excellent/very good/good” health, while 95.0% of children in our sample had medical insurance. Concerning nativity, 78.4% of children in our sample had been born in the U.S., as had 20.8% of parents in our sample. Furthermore, English was the primary language used at home for 61.1% of families in our sample. Median age of parents in our study was 44.2 years and children 10 years; 50.9% of children in the sample were girls.

Results from multivariate analysis showed the hypothesized model to differ significantly from the null (Wald’s χ^2^ = 71.15, *p* < 0.01; see Table 2). Specifically, results indicated that residence in a safe neighborhood was associated positively with the likelihood that a child in the sample was in “excellent/very good/good” health (OR = 2.35; *p* < 0.05). Living in rundown neighborhoods and experiencing racial/ethnic discrimination, however, showed no association with children’s health in this study. Only two of the tested social structural variables exhibited a significant relationship with our outcome variable—both in positive direction: family income-to-poverty ratio (OR = 1.01, *p* < 0.01) and receipt of TANF (OR = 275.79, *p* < 0.05). In our study, while single motherhood (OR = 0.09, *p* < 0.01) was associated negatively with child health, child’s “excellent/very good/good” health was associated positively with family cohesiveness (OR = 2.17, *p* < 0.01) and family support (OR = 15.20, *p* < 0.05). Emotional support (for parents) from healthcare professionals, peer/religious groups, and neighbors did not demonstrate significant effect on child’s health in our study. A similar lack of significant association was observed for receipt of SNAP, for acculturation factors, and for the demographic characteristics age (child’s as well as parent’s) and gender.

## 4. Discussion

Our study showed over 98% of ethnic Asian children in the sample to be in “excellent/very good/good” health. Moreover, our multivariate analysis findings showed child health to have associations in positive direction with safe neighborhood, family income-to-poverty ratio, receipt of TANF, family cohesiveness, and family support; and associations in negative direction with single mother. No other variables showed significant associations with children’s health.

The present study found that the majority of ethnic Asian children in the sample were in “excellent/very good/good” health, a proportion comparable to prior published results [6,7,9]. Our findings, moreover, partially supported our first hypothesis, that the health of ethnic Asian children would be associated positively with safe neighborhoods, family income, parents’ educational attainment, parent’s employment, family support, social support, parents’ health, medical insurance coverage, TANF or SNAP participation, parents’ U.S. birth, children’s U.S. birth, and families’ English proficiency. In the present study, we observed the health of ethnic Asian children to be better with residence in safe neighborhoods. We also observed a relationship in positive direction between their health and their family income, a finding that supports results from prior studies of children in the general population [2,3,4,13,35,36,37]. Our study found that receiving TANF was linked to better child health. Unlike a study focused on several cities [38], however, our research found no significant association between ethnic Asian children’s health and family participation in SNAP. Thus our overall findings imply that relatively high family incomes and safe neighborhoods facilitate ethnic Asian children’s good health and that assistance from TANF has a similar effect among low-income families. Examining our data even more closely showed that merely 9.0% of families in our sample had incomes below federal poverty level; of those, 3.3% participated in TANF, 21.5% participated in SNAP, and 62.8% had an employed parent. In other words, no TANF or SNAP assistance seems to be sought by the majority of ethnic Asian working-poor families.

In contrast, in line with other prior results for ethnic Asian children [30,31], we found no association between child health and parent’s educational background. Our findings additionally showed child health to be associated positively with family cohesiveness and family support. Professionals’ provision of emotional support for parents did not change health among the ethnic Asian children in our sample, however; this finding parallels at least one earlier published result [45]. Support for parents from peer-support or religious groups, and from neighbors, similarly lacked demonstrable effect on child health in this study. Such findings suggest that Asian families rely heavily on their relatives and close friends for support, regardless of parents’ educational backgrounds.

Like another study [31], our study observed no association between ethnic Asian children’s health and the birthplace of their parents or their own birthplace. *Unlike* other studies, though—all of them having small samples, our study indicated no significant association between English proficiency and ethnic Asian children’s health [8,51]. While many ethnic Asian families maintain the culture of origin and have difficulties of cultural adjustment [57], our findings found no association between children’s health and acculturation.

Our study findings also partially supported our second hypothesis, that the health of ethnic Asian children would be associated negatively with rundown neighborhoods, experiences of racial/ethnic discrimination, and single-parent households. The negative relationship observed, in this study, between child health and households headed by single mothers in particular tends to confirm some prior studies with children in the general population [36,43]. It appears that single mothers can find caring for children to be difficult and stressful; that our outcome (child health) was significantly linked to family support implies that such mothers need a great deal of support from their relatives and close friends. At the same time, we did not observe any significant links, here, between child health and rundown neighborhoods or discrimination experiences. Moreover, close examination of the data revealed that the interaction term between single mother and safe neighborhood (OR = 0.02, *p* < 0.05) yielded a negative association with child health, while interactions terms between single mother and family income-to-poverty ratio, receipt of TANF, family cohesiveness, and family support yielded no significant associations. In fact, many ethnic Asian single mothers report experiencing stigmatization and receiving meager support from family members and peers [58,59,60]. Such findings suggest that single mothers can have difficulty ensuring their children’s health, even with residence in a safe neighborhood.

Our present study had several limitations. The first is inherent in the analyzed data’s cross-sectional nature. Because we used cross-sectional data, any causal or directional relationships between tested variables merely reflected theoretical assumptions of the multiple disadvantage model. Second, preliminary analyses we conducted exhibited singularity while examining parent’s length of residence in U.S., family mental health problems, and family substance use, impeding our application of the full conceptual framework of the multiple disadvantage model. Third, our present study could not include length of parent’s residence in U.S. nor parent’s participation Medicaid. Without these two variables, our analysis was unable to examine underlying reasons—such as cultural influences and states’ Medicaid policies—for the sampled families’ low rate of participation in public assistance. It is important to remember that these limitations, necessitate a cautious approach to any generalization of our findings.

## 5. Conclusions

Applying the conceptual framework provided by the multiple disadvantage model identified several factors in the health of ethnic Asian children in the U.S., each of those factors describing social disorganization, social structure, or social integration. Most important is the implication from our analysis that interventions would most benefit ethnic Asian families who are impoverished and living in unsafe neighborhoods. Social work professionals should advocate community policing and neighborhood watch groups in ethnic Asian communities, since these constitute effective means of promoting neighborhoods’ safety [61,62]. Collaboration of social workers with public-health professionals on one hand and law enforcement professionals on the other should also help create safe Asian neighborhoods [24].

Our findings confirm that TANF assistance is associated with better health among ethnic Asian children. It is thus crucial to raise low-income ethnic Asian families’ awareness of TANF and similar programs, especially concerning program eligibility. Public education events organized by community centers and other public-welfare organizations active in Asian communities are one option. To improve health among children of ethnic Asian families headed by single-mothers, social workers and public-health advocates should help the women establish strong social bonds, for example supportive relationships with their relatives and friends. Additionally, those in the helping professions should consistently demonstrate respect for and understanding of Asian cultural assumptions and values concerning children’s health and well-being.

Future research in the same vein as the present study might investigate how ethnic Asian children’s health is impacted by their parents’ participation in public medical insurance programs. As well, many ethnic Asian single mothers may have mood disorders [59], so future research might explore whether and how stress-related mental health or substance use problems they exhibit affect their children’s health. Finally, future research might productively involve longitudinal data accommodating analysis of the full conceptual framework of the multiple disadvantage model.

## Figures and Tables

**Table 1:** Descriptive statistics of ethnic Asian children (*n* = 1350).

	Frequency	Percent	M	IQR
Outcome variable				
Child health (excellent/very good/good)	1331	98.6	N/A	N/A
(fair/poor)	19	1.4	N/A	N/A
Explanatory variables				
Rundown neighborhood (yes)	350	25.9	N/A	N/A
(no)	1000	74.1	N/A	N/A
Racial discrimination (yes)	84	6.2	N/A	N/A
(no)	1266	93.8	N/A	N/A
Safe neighborhood	N/A	N/A	4	1
Parent educational attainment	N/A	N/A	7	8
Employed parent (yes)	1106	81.9	N/A	N/A
(no)	244	18.1	N/A	N/A
Family income-to-poverty ratio (%)	N/A	N/A	390	199
Receipt of TANF (yes)	16	1.2	N/A	N/A
(no)	1334	98.8	N/A	N/A
Receipt of SNAP (yes)	58	4.3	N/A	N/A
(no)	1292	95.7	N/A	N/A
Single mother (yes)	77	43.7	N/A	N/A
(no)	1273	56.3	N/A	N/A
Family cohesiveness	N/A	N/A	7	2
Family support (yes)	700	51.9	N/A	N/A
(no)	650	48.1	N/A	N/A
Professional support (yes)	221	16.4	N/A	N/A
(no)	1129	83.6	N/A	N/A
Peer/religious group support (yes)	255	18.9	N/A	N/A
(no)	1095	81.1	N/A	N/A
Neighbor support	N/A	N/A	10	3
Parent health (excellent/very good/good)	1303	96.5	N/A	N/A
(fair/poor)	47	3.5	N/A	N/A
Insured (yes)	1282	95.0	N/A	N/A
(no)	68	5.0	N/A	N/A
Parent born in U.S. (yes)	281	20.8	N/A	N/A
(no)	1069	79.2	N/A	N/A
Child born in U.S. (yes)	1059	78.4	N/A	N/A
(no)	291	21.6	N/A	N/A
Speaks English at home (yes)	825	61.1	N/A	N/A
(no)	525	38.9	N/A	N/A
Parent age (years)	N/A	N/A	44	10
Child age (years)	N/A	N/A	10	9
Girl	687	50.9	N/A	N/A
Boy	663	49.1	N/A	N/A

Note: M = median; IQR = interquartile range; N/A = not applicable.

**Table 2:** Logistic regression results on ethnic Asian child health (excellent/very good/good) (*n* = 1350).

Variables	OR	RSE	90% Confidence-Interval
Rundown neighborhood (no)	0.73	0.57	0.20–2.65
Racial discrimination (no)	2.27	2.35	0.41–12.43
Safe neighborhood	2.35 *	1.17	1.03–5.33
Parent educational attainment	0.76	0.13	0.57–1.00
Employed parent (no)	0.76	0.84	0.12–4.71
Family income-to-poverty ratio	1.01 **	0.00	1.00–1.01
Receipt of TANF (no)	275.79 *	739.56	3.35–22,708.61
Receipt of SNAP (no)	0.27	0.29	0.05–1.59
Single mother (no)	0.09 **	0.08	0.02–0.40
Family cohesiveness	2.17 **	0.49	1.50–3.14
Family support (no)	15.20 *	20.20	1.71–135.23
Professional support (no)	0.35	0.45	0.04–2.90
Peer/religious group support (no)	0.28	0.38	0.03–2.58
Neighbor support	1.01	0.16	0.78–1.30
Parent health (fair/poor)	4.35	4.77	0.72–26.40
Insured (no)	3.74	3.04	0.98–14.26
Parent born in U.S. (no)	0.74	0.76	0.14–4.01
Child born in U.S. (no)	1.31	1.10	0.33–5.21
Speaks English at home (no)	2.52	2.35	0.54–11.66
Parent age	0.97	0.07	0.86–1.09
Child age	0.97	0.10	0.82–1.16
Girl (boy)	0.39	0.24	0.14–1.08
Wald’s χ^2^ =	71.15 **		

Notes: ** *p* < 0.01; * *p* < 0.05; OR = odds-ratios; RSE = robust standard errors; reference groups are in parentheses.

## Data Availability

This study employed solely public-use data.
